# Supplementary material for: Unveiling immune interference: how the dendritic cell response to co-infection with Aspergillus fumigatus is modulated by human cytomegalovirus and its virokine CMVIL-10
Source: mBio. 2025 Sep 22;16(11):e01541-25. doi: 10.1128/mbio.01541-25 (PMC12607784; doi:10.1128/mbio.01541-25)
Supplement: Supplemental Material — Supplemental methods, tables, and figures. [file mbio.01541-25-s0001.docx]

**Unveiling immune interference: How the dendritic cell response to co-infection with *Aspergillus fumigatus* is modulated by human cytomegalovirus and its virokine _CMV_IL-10**

Supplementary Materials

Supplementary Methods:

Generation of monocyte-derived dendritic cells (moDCs)

In brief, peripheral blood mononuclear cells (PBMCs) were obtained from healthy adults using leukoreduction chambers, a byproduct of plateletpheresis donations. Monoclonal cells were isolated by gradient-density centrifugation with Ficoll solution (density 1.077 g/mL) and quantified using a Vi-Cell XR counter. CD14^+^ monocytes were isolated from PBMCs by positive selection using human CD14 Micro Beads (Miltenyi Biotec) according to the manufacturer’s instruction. To generate moDCs, monocytes were cultured in CellGenix GMP DC Medium (1×10⁶ cells/mL, Sartorius) supplemented with 120 µg/ml gentamicin (Refobacin, Merck), GM-CSF (1000 U/mL, Miltenyi Biotec), and IL-4 (1000 U/mL, Miltenyi Biotec). After 6 days of culture at 37 °C and 5% CO_2_, the differentiated moDCs were harvested and carefully detached from the plate using a cell scraper. After centrifugation for 10 min at 300 ×*g*, moDCs were resuspended in CellGenix (2×10⁶ cells/mL) for subsequent infection.

RNA sequencing statistical analysis and analytic models

Differential gene expression analysis was done using DESeq2 (v1.46.0, R version 4.4.2) considering a multiple-test adjusted p ≤ 0.05 and log2 (fold-change) ≥ 1 as cut-offs for significance. Given donor heterogeneity, DESeq2 statistical designs were created to control for donor origin (design = ~ donor + condition) and different pairwise condition differences, unless otherwise noted. Additional DESeq2 designs to control for single infections when testing co-infection (CoI) with _CMV_IL-10 competent TB40 HCMV strains (design = ~ donor + TB40 + AF + CoI) _CMV_IL-10 incompetent Δ*UL111A* strain (design = ~ donor + Δ*UL111A* + AF + CoI) or both (design = ~ donor + TB40 + Δ*UL111A* + AF + CoI) were applied, where all controlling factors except donor were one-hot encoded (control sample = yes or no, TB40 sample = yes/no, etc.).

Multiplex assays for cytokine secretion

ProcartaPlex assay kits (ThermoFisher Scientific) containing the following analytes were used to quantify cytokine and chemokine concentration in the supernatant of infected moDCs (IFN-α, IFN-γ, IL-1α, IL-1β, IL-2, IL-6, IL-8, IL-10, IL-23, CXCL10, CXCL11, CCL2, CXCL9, CCL3, CCL4, CCL20, CCL5, and TNF-α (Figure S1, Figure 4B) or IFN-α, IFN-β, IFN-γ, IL-1α, IL-1β, IL-2, IL-6, IL-8, IL-10, IL-12p70, IL-17A, IL-23, CXCL10, CXCL11, CCL5 and TNF-α (Figure 5B).

IncuCyte time-lapse imaging

In brief, well plates were imaged hourly in an IncuCyte Zoom HD/2CLR time-lapse microscopy system (Sartorius, Göttingen, Germany) equipped with an IncuCyte Zoom 10× Plan Fluor objective (Sartorius, Göttingen, Germany) for a period of 18 h. Acquisition time for the red channel was 300 ms. The following parameters were used for NeuroTrack analysis: neurite coarse sensitivity, 10; neurite fine sensitivity, 0.75; neurite width, 4 µm. Neurite length [mm/mm^2^] and numbers of branch points [1/mm^2^]) were compared to an “*A. fumigatus* only” control without moDCs and/or HCMV/ _CMV_IL-10

Table S1: Primers used for quantitative real-time PCR

| CXCL8 | fw | GTTTTTGAAGAGGGCTGAG |
| --- | --- | --- |
|  | rev | TTTGCTTGAAGTTTCACTGG |
| VEGFA | fw | AATGTGAATGCAGACCAAAG |
|  | rev | GACTTATACCGGGATTTCTTG |
| IRF5 | fw | CTCAGCCCTACAAGATCTAC |
|  | rev | CTGCACCAAAAGAGTAATCC |
| CD40 | fw | CTGGCACTGTACGAGTGAGG |
|  | rev | AAGACCAGCACCAAGAGGATG |
| CXCL9 | fw | AGGTCAGCCAAAAGAAAAAG |
|  | rev | TGAAGTGGTCTCTTATGTAGTC |
| LILRB1 | fw | GAATGAGGAGAAAGCAAGAAG |
|  | rev | TGAGCTTGATGTAAATGTGC |
| GAPDH | fw | GGACTGAGGCTCCCACCTTT |
|  | rev | GCATGGACTGTGGTCTGCAA |

Table S2: Flow cytometry antibodies and reagents

| **Antibodies** | **Source** | **Identifier** |
| --- | --- | --- |
| Anti-human CD1c (L161) BrilliantViolet650 | Biolegend, San Diego, CA, USA | Cat#331542 |
| Anti-human CD14 (REA599) Viogreen | Miltenyi Biotec, Bergisch Gladbach, Germany | Cat#130-110-583 |
| Anti-human CD86 (REA968) APC-Vio770 | Miltenyi Biotec, Bergisch Gladbach, Germany | Cat#130-116-163 |
| Anti-human TLR3 (TLR 3.7) APC * | Miltenyi Biotec, Bergisch Gladbach, Germany | Cat#130-096-885 |
| Anti-human CD209 (REA617) PE-Vio770 | Miltenyi Biotec, Bergisch Gladbach, Germany | Cat#130-128-219 |
| Anti-human cGAS (E5V3W) Alexa Fluor 647 * | Cell Signaling, Beverly, MA, USA | Cat#43398S |
| Anti-human hSTING (723505) PE * | R&D Systems, Minneapolis, MN, USA | Cat#IC7169P |
| Anti-human IRF3 (482205) Alexa Fluor 750 * | R&D Systems, Minneapolis, MN, USA | Cat#FAB4019S |
| Anti-human IRF5 Alexa Fluor 700 * | R&D Systems, Minneapolis, MN, USA | Cat#IC4508N |
| Viobility 405/452 | Miltenyi Biotec, Bergisch Gladbach, Germany | Cat#130-110-205 |

Asterisks (*) indicate markers, which have been stained intracellularly.


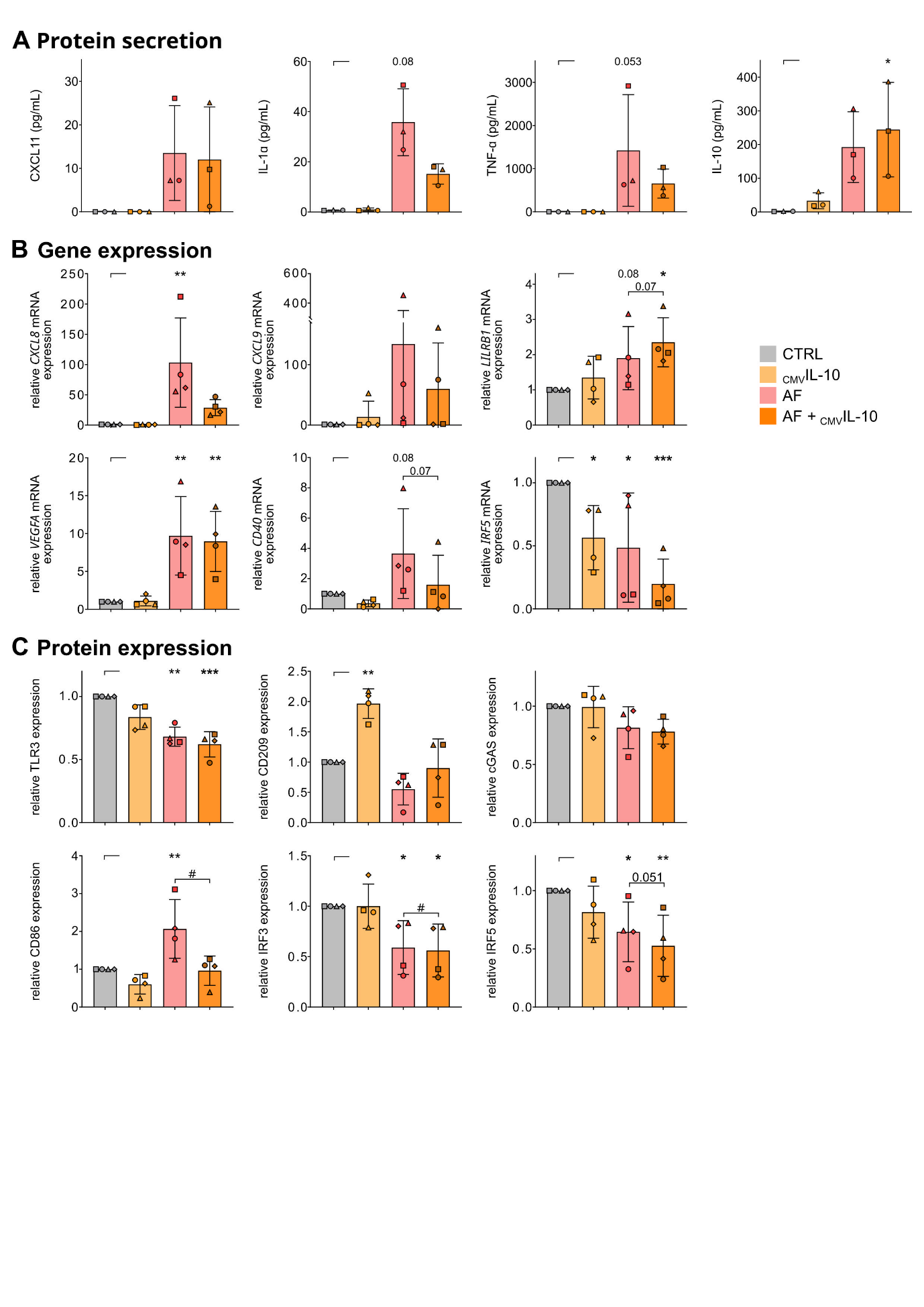


**Figure S1 (caption on following page)**

**Figure S1: Recombinant _CMV_IL-10 does not significantly alter *A. fumigatus*-induced responses of moDCs at both mRNA and protein levels.**

Cytokine/chemokine release (A), relative mRNA expression (B), and flow cytometric analysis (C) of naïve moDCs (CTRL) and those pre-incubated with _CMV_IL-10 for 24 h, with or without subsequent *A. fumigatus* (AF) infection for another 9 h. (A) N = 3 independent donors. Friedman test with Dunn’s multiple comparisons test versus CTRL (asterisks). (B-C) N = 4 independent donors. Repeated measures one-way analysis of variance with Dunnett’s post-hoc test versus CTRL (asterisks). In addition, single AF infection was compared to co-stimulation (AF + _CMV_IL-10) using paired t-test (hash signs). (A-C) Columns and error bars indicate means and standard deviations, respectively. */# p < 0.05, **/## p < 0.01, ***/### p < 0.001

**Figure S2 (caption on following page)**

**Figure S2. Efficiency of moDC infection by HCMV.**

(A) moDCs were (pre)-exposed for 0 h, 24 h, or 72 h to EGFP-expressing TB40 or Δ*UL111A* or left unchallenged (CTRL). Cells were then co-cultured with *A. fumigatus* (AF) or cultured without fungal exposure for another 9 h, while IncuCyte imaging was performed. Images from one representative donor (out of four) are shown. (B) Flow cytometric analysis of moDCs (CTRL) (pre)-exposed to TB40 or Δ*UL111A* for 0 h, 24 h, or 72 h, with or without subsequent AF exposure. The percentage of GFP^+^ moDCs was determined as a marker of infection efficiency. N = 4 independent donors. Columns and error bars indicate means and standard deviations, respectively.


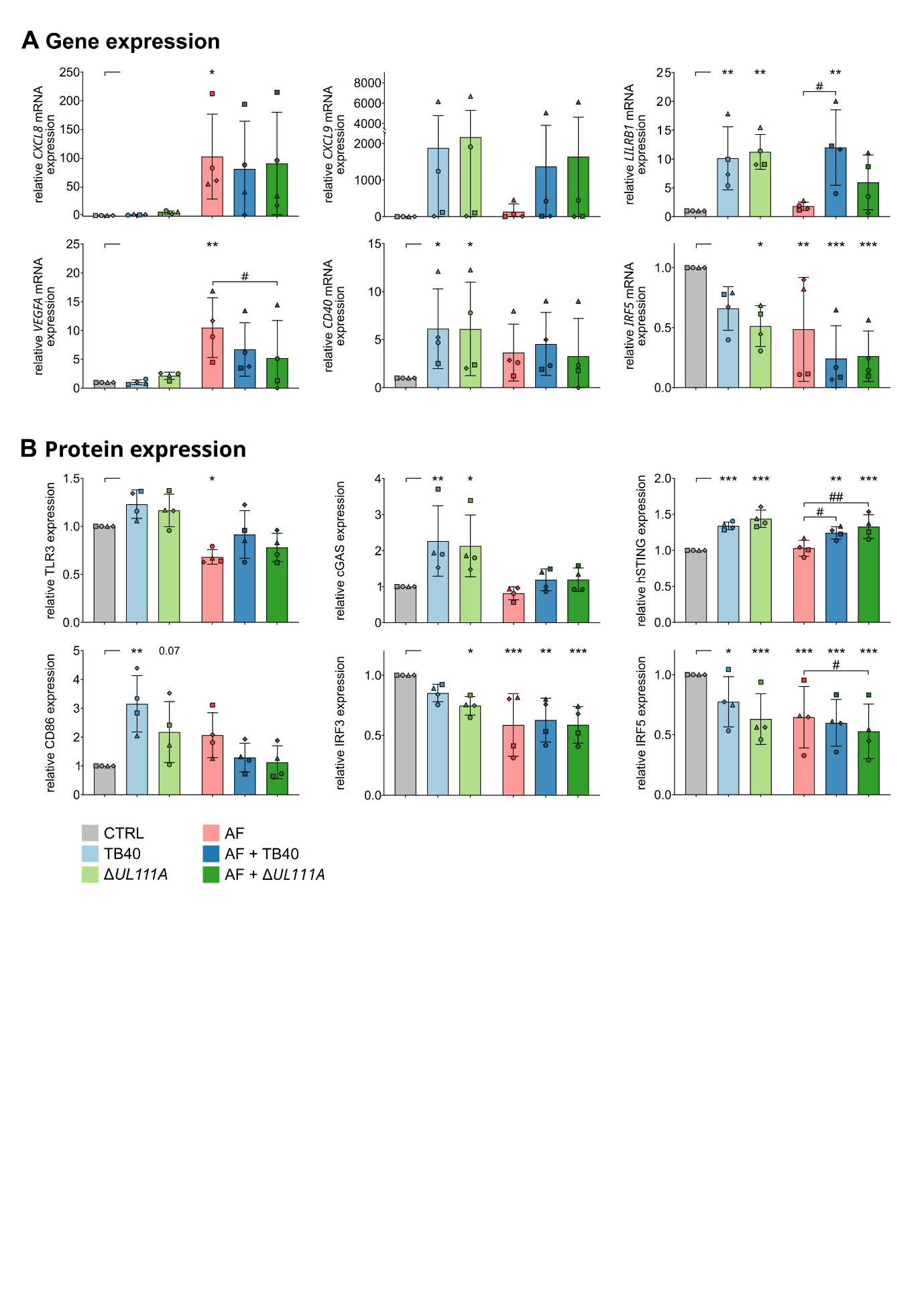
**Figure S3 (caption on following page)**

**Figure S3: _CMV_IL-10 modulates the antiviral response of moDCs but has minimal effects on HCMV-mediated suppression of antifungal responses at both mRNA and protein levels.**

(A) Relative mRNA expression of moDCs confronted with TB40, Δ*UL111A*, and/or *A. fumigatus* (AF), either individually (TB40, Δ*UL111A*, AF) or in combination (AF + TB40, AF + Δ*UL111A*). (B) Flow cytometric analysis of moDCs pre-incubated with TB40 or Δ*UL111A* for 24 h prior to culture with or without AF infection for another 9 h. (A-B) N = 4 independent donors. Columns and error bars indicate means and standard deviations, respectively. Repeated measures (RM) one-way analysis of variance (ANOVA) and Dunnett’s post-hoc test versus “CTRL”, i.e., uninfected moDCs (asterisks). In addition, single AF infection was compared to co-infection (AF + TB40, AF + Δ*UL111A*) using RM one-way ANOVA and Dunnett’s post-hoc test versus “AF” (hash signs). */# p < 0.05, **/## p < 0.01, ***/### p < 0.001.


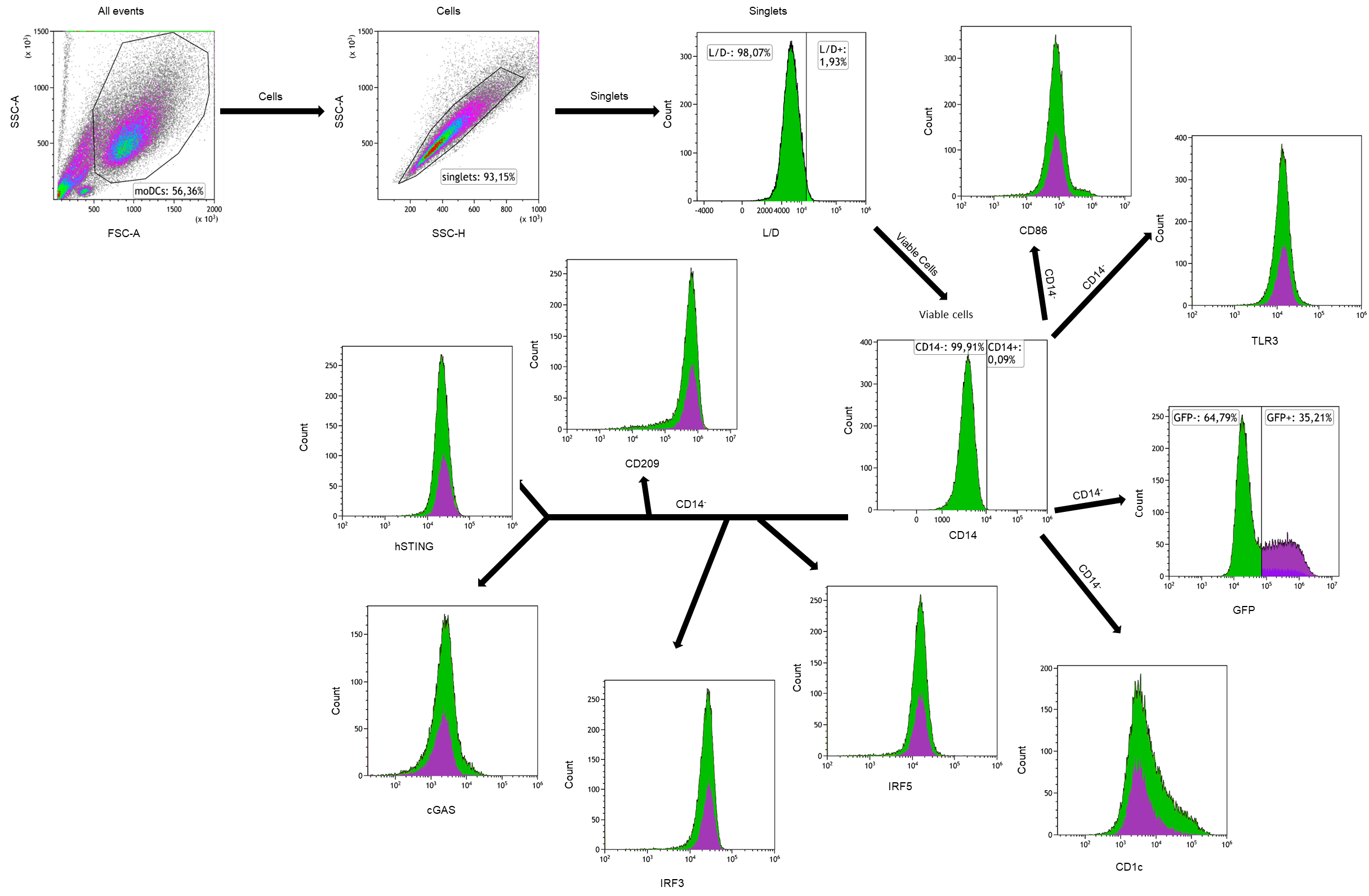


**Figure S4. Gating strategy to study efficiency of moDC infection by HCMV, moDC maturation and activation.**

MoDCs were selected according to their forward and side scattering. Single cells were then gated based on SSC-A/SSC-H properties. Dead cells were excluded by Live/Dead staining. The specificity of moDC gating was enhanced by exclusion of CD14^+^ cells. Among CD14^-^ cells (moDCs), those infected by HCMV were identified by GFP positivity (purple histograms). Mean fluorescence intensities (MFI) for cell surface markers (CD1c, CD86,CD209) and intracellular markers (TLR3, IRF3, IRF5, cGAS, hSTING) were determined for both uninfected (dark green) and HCMV-infected (purple) CD14^-^ moDCs.
